# *Sphingomonas mucosissima* Bacteremia in Patient with Sickle Cell Disease

**DOI:** 10.3201/eid1501.080465

**Published:** 2009-01

**Authors:** Emmanouil Angelakis, Véronique Roux, Didier Raoult

**Affiliations:** Faculté de Médecine et de Pharmacie–Université de la Méditerranée, Marseille, France

**Keywords:** Sphingomonas mucosissima, bacteremia, sickle cell disease, letter

**To the Editor**: The genus *Sphingomonas* was proposed by Yabuuchi et al. in 1990 (*1*) and amended by Takeuchi et al. in 1993 (*2*). It now has been subdivided into 4 separate genera: *Sphingomonas sensu stricto*, *Sphingobium*, *Novosphingobium,* and *Sphingopyxis*. The bacteria of the genus *Sphingomonas* are yellow-pigmented, nonfermenting, gram-negative bacilli with a single polar flagellum; they are widely distributed in the natural environment, especially in water and soil (*3*). These bacteria are characterized by the presence of a unique sphingoglycolipid with the long-chain base—dihydrosphingosin, ubiquinone 10 (Q-10), and 2-hydroxymyristic acid (2-OH C14:0)—and the absence of 3-hydroxy fatty acids (*4*). *S. mucosissima* was isolated and identified in 2007 by Reddy and Garcia-Pichel from biologic soil crust samples collected from sandy arid soil in the US Colorado Plateau (*5*). *Sphingomonas* spp. are opportunistic pathogens and have recently been implicated in a variety of community-acquired and nosocomial infections, considered to originate from contaminated hospital equipment or manipulation of some medical devices (*3*). The survival of *Sphingomonas* spp. in indoor dust particles as aerosols and their resistance to many disinfecting and toxic chemicals may explain their ability to colonize medical devices such as mechanical ventilators, catheters, and bronchofiberoscopes (*6*). In the past few years, these organisms, in particular *S. paucimobilis*, have been implicated in a variety of community-acquired and nosocomial infections.

We report a case of *S. mucosissima* bacteremia in a patient with sickle cell disease. In February 2008, a 17-year-old woman with homozygous sickle cell anemia was hospitalized when her condition suddenly became worse. The patient had undergone a splenectomy in 1992 and a cholecystectomy in February 2007. Four days after admission, she had a fever of 38.7°C. Two aerobic blood specimens, drawn on the fifth day of her hospitalization, yielded gram-negative bacilli after a 24-hour incubation. The gram-negative bacilli were positive for catalase and oxidase but remained unidentified by API 20NE strip (bioMérieux, Marcy l’Etoile, France). MICs of antimicrobial drugs were determined for the gram-negative bacilli by using an Etest assay (AB BIODISK, Solna, Sweden) on Mueller-Hinton medium. MICs were 1 μg/mL for cefotaxime, 1 μg/mL for amoxicillin–clavulanic acid, 2–3 μg/mL for vancomycin, 0.064 μg/mL for imipenem, 4–5 μg/mL for ceftazidime, 1 μg/mL for amikacin, 3 μg/mL for ciprofloxacin, and 0.047 μg/mL for trimethoprim-sulfamethoxazole.

DNA was extracted from 1 colony by using a QIAamp Tissue kit (QIAGEN, Hilden, Germany) as described by the manufacturer. A 16S rDNA sequence was obtained (1,410 bp) by using the fD1 (5′-AGAGTTTGATCCTGGCTCAG-3′) and rP2 (5′-ACGGCTACCTTGTTACGACTT-3′) primer pair (*7*,*8*). Using BLAST version 2.2.9 software (www.ncbi.nlm.nig.gov/BLAST), we determined that this sequence showed 98% similarity with the 16S rDNA sequence of *S. mucosissima* (GenBank accession no. AM229669). A phylogenetic neighbor-joining tree resulting from comparison of sequences of the 16S rDNA genes of *Sphingomonas* spp. was made with the MEGA 3.1 software (www.megasoftware.net). This analysis confirmed that the isolate belonged to *S. mucosissima*.

Initial treatment of intravenous administration of ceftriaxone was begun. The fever resolved after 1 day and the patient’s condition improved. Treatment was stopped after 5 days, and the patient remained apyretic. Two *S. mucosissima* isolates were recovered from 2 different blood-culture samples drawn 24 hours apart, which suggests that *S. mucosissima* was not just a transient organism but indeed was responsible for the patient’s septicemia. Phenotypic identification of the gram-negative bacilli failed because the definite bacterial species *S. mucosissima* was not included in the API database (http://industry.biomerieux-usa.com/industry/food/api/apiweb.htm) used for the phenotypic identification. However, the isolates’ biochemical characteristics were consistent with those previously reported for *S. mucosissima* (*5*) (Table). Final identification was achieved by comparing the almost complete 16S rDNA sequence with homologous sequences deposited in GenBank.

**Table:** Biochemical characteristics of the previously reported *Sphingomonas mucosissima* isolate (AM229669) and the isolate from this study

Characteristic	*S. mucosissima*	Isolate from this study
Biochemical characteristics		
Oxidase	+	+
Catalase	+	+
Phosphatase	+	+
β-galactosidase	–	–
Gelatinase	–	–
Nitrate reduction	–	–
Assimilation of carbon compounds		
Alanine	+	+
Glucose	+	+
Glutamic acid	–	–
Mannitol	–	–
Sucrose	+	+

We believe that the patient’s intravenous catheter was the source of the infection because she did not have wound infections and cultures of her urine were negative for infectious agents. Antimicrobial drug treatment, selected on the basis of an in vitro *S. mucosissima* susceptibility profile, facilitated the patient’s recovery. This case report illustrates that the pathogenic potential of *S. mucosissima* should be considered in diagnosis in such cases because the organism can cause bacteremia in patients, primarily in those with underlying debilitating conditions and those who have undergone medical interventions.
